# Sex‐specific hepatic effects of sweetened alcohol consumption and tannic acid intervention in adolescent rats

**DOI:** 10.14814/phy2.70992

**Published:** 2026-06-30

**Authors:** Toluwase E. Olanipekun, Oladiran I. Olateju, Monica Gomes, Ashmeetha Manilall, Kennedy H. Erlwanger

**Affiliations:** ^1^ Department of Physiology, School of Biomedical Sciences, Faculty of Health Sciences University of the Witwatersrand Johannesburg South Africa; ^2^ Department of Anatomical Sciences, School of Biomedical Sciences, Faculty of Health Sciences University of the Witwatersrand Johannesburg South Africa

**Keywords:** adolescents, alcohol drinking, fructose, liver, oxidative stress, sex differences, Sprague–Dawley, tannic acid

## Abstract

This study investigated the combined effects of alcohol and sugar, termed sweetened alcohol consumption (SAC), and tannic acid (TA) intervention on hepatic health in adolescent rats. Sixty‐four male and female 42‐day‐old Sprague–Dawley rats were assigned to control, TA (50 mg/kg), SAC (10% ethanol +20% fructose), or SAC+TA groups and treated for 10 weeks using a voluntary gelatine‐based model. Growth parameters, feed intake, visceral fat, fasting glucose, insulin, HOMA‐IR, lipid profiles, and hepatic outcomes, including mass, TBARS, gene expression, and histological features, were assessed. SAC reduced feed intake without affecting body mass. Females exposed to SAC showed increased visceral fat, elevated hepatic TBARS, reduced hepatocyte density, and more pronounced steatosis, indicating oxidative and structural susceptibility. In contrast, males exhibited increased HDL‐cholesterol with reduced hepatic TBARS, suggesting sex‐specific differences in hepatic metabolic adaptation. TA co‐treatment prevented the SAC‐associated increase in hepatic TBARS but did not preserve hepatocyte density or alter steatosis in females. Glucose homeostasis, insulin resistance, triglycerides, and hepatic expression of *CYP2E1*, *SREBP‐1*, *IL‐10*, *NF‐κB1*, and *TNF‐α* were unaffected across groups. Overall, SAC induces sex‐dependent hepatic alterations during adolescence, with females showing heightened vulnerability. TA did not produce a measurable additional benefit when combined with SAC under the conditions used.

## 1 INTRODUCTION

The global increase in adolescent metabolic syndrome represents a major emerging public health concern (Noubiap et al., 2022). Characterized by visceral obesity, insulin resistance, dyslipidaemia, hyperglycaemia, and oxidative stress, it reflects systemic metabolic dysfunction that substantially contributes to the burden of noncommunicable disease (Papaioannou et al., 2022). Unhealthy lifestyle habits, particularly poor diet and physical inactivity, are increasingly common among adolescents and strongly linked to metabolic disturbances (Al‐Qawasmeh & Tayyem, 2018; Lin et al., 2019). Among the resulting complications, liver disease has gained prominence and now ranks as the 11th leading cause of global mortality (Devarbhavi et al., 2023). As the central organ responsible for nutrient metabolism and detoxification, the liver is highly vulnerable to chronic exposure to dietary and environmental insults (Acharya et al., 2021).

Chronic consumption of alcohol and sugar, particularly among adolescents, imposes considerable metabolic stress on the liver, contributing to the development of both alcoholic liver disease (ALD) and metabolic‐related steatotic liver diseases (Sharma et al., 2021). Recent advances in understanding the interplay between metabolic dysfunction and alcohol consumption have prompted a reconceptualisation of liver disease within a broader continuum encompassing metabolic dysfunction–associated steatotic liver disease (MASLD) and the newly defined metabolic dysfunction–associated alcohol‐related liver disease (MetALD) (Ciardullo & Perseghin, 2024). These revisions mark a significant shift from earlier terminology such as non‐alcoholic fatty liver disease (NAFLD) and metabolic‐associated fatty liver disease (MAFLD), aligning disease classification more closely with underlying metabolic and lifestyle determinants (Ciardullo & Perseghin, 2024).

The dual burden of alcohol and sugar consumption is particularly concerning in adolescents. Recent reports indicate a steady rise in alcohol use among this age group (Farnia et al., 2024), paralleled by persistently high intake of sugar‐sweetened beverages (Morgan et al., 2021). Exposure to these substances during early life increases vulnerability to metabolic dysfunction and liver disease in adulthood. The convergence of these risk factors during a critical developmental window may exacerbate metabolic stress and accelerate the onset of chronic disease, consistent with the observed rise in metabolic disorders among young adults (Bradwisch et al., 2020; Calcaterra et al., 2023).

The metabolic impact of alcohol and sugar extends well beyond hepatic dysfunction. Alcohol disrupts nutrient absorption and intestinal integrity, leading to deficiencies that impair physiological development (Butts et al., 2023). Similarly, excessive intake of dietary sugars, particularly high‐fructose sweeteners, alters lipid metabolism, promotes fat deposition, and compromises glucose regulation (Franczyk et al., 2021). Collectively, these disturbances contribute to obesity, insulin resistance, and systemic inflammation (Calcaterra et al., 2023). When consumed together, as in sweetened alcoholic beverages, their detrimental metabolic effects may be synergistically amplified (Alwahsh et al., 2014), yet this interaction remains poorly characterized in adolescents. Current treatment approaches are often inadequate due to the multifactorial nature of these metabolic disturbances. Conventional pharmacological therapies typically target individual components of metabolic syndrome (Makhoba et al., 2020), necessitating polypharmacy that increases treatment cost, side effects, and reduces adherence (Elkordy et al., 2021; Lillich et al., 2021). These challenges have intensified interest in holistic, cost‐effective, and accessible alternatives for preventing or managing metabolic disorders (Xu et al., 2021).

Phytochemicals, bioactive compounds derived from plants, are emerging as promising therapeutic agents for the prevention and management of metabolic disorders (Xu et al., 2021). Generally regarded as safe, affordable, and widely accessible, these compounds provide multifaceted physiological benefits with fewer side effects than conventional pharmacological treatments (Fang et al., 2020). Among them, tannic acid, a naturally occurring polyphenol abundant in fruits, vegetables, and plant‐derived beverages, has attracted growing interest for its antioxidant, anti‐inflammatory, anti‐obesity, and anti‐diabetic properties (Jing, Xiaolan, et al., 2022; Türkan et al., 2019). These attributes make tannic acid a compelling candidate for targeting the interconnected metabolic disturbances associated with excessive sugar and alcohol consumption.

Experimental evidence supports tannic acid's therapeutic potential in preventing or alleviating metabolic and hepatic disorders (Das et al., 2022). It exerts broad biological effects by modulating inflammatory pathways, reducing oxidative stress, and improving lipid and glucose metabolism (Jing, Niu, et al., 2022). In murine models, tannic acid combined with vitamin E in nanoparticle formulations effectively prevented alcohol‐induced liver injury (Nag et al., 2020), while other studies demonstrated its capacity to inhibit the progression of NAFLD through epigenetic regulation, including histone acetylation (Chung et al., 2019). Collectively, these findings indicate tannic acid may provide a multi‐targeted intervention for improving metabolic health and mitigating diet‐ or alcohol‐induced hepatic damage.

Despite promising evidence, significant knowledge gaps remain regarding how tannic acid mitigates the combined metabolic effects of sugar and alcohol, particularly in the context of the growing consumption of sweetened alcoholic beverages among adolescents. Moreover, sex‐based differences in vulnerability to metabolic and hepatic dysfunction are insufficiently explored. Hormonal and physiological variations between males and females may differentially influence disease progression and responsiveness to intervention, highlighting the importance of sex‐specific research. Addressing these gaps is critical for developing effective and targeted preventive strategies. Exploring tannic acid's modulatory effects on SAC across sexes may therefore yield valuable insights into its therapeutic potential, making this research both timely and necessary. Therefore, the present study evaluated the protective effects of tannic acid supplementation against metabolic and hepatic disturbances induced by SAC in adolescent male and female rats. It was hypothesized that tannic acid would attenuate the metabolic alterations and hepatic dysfunction associated with this combined dietary insult, thereby providing insight into its potential as an early intervention strategy for mitigating metabolic risk in vulnerable adolescent populations.

## 2 MATERIALS AND METHODS

### 2.1 Ethical approval and study location

The study was granted ethical clearance (AREC/2022/11/06C) from the Animal Research Ethics Committee at the University of the Witwatersrand, Johannesburg. All experiments were carried out at the Wits Research Animal Facility (WRAF) at the University of the Witwatersrand, in accordance with the South African National Standard for animal care and use for scientific purposes (SANS 10386, 2021). ARRIVE 2.0 guidelines for reporting research have been followed (Percie du Sert et al., 2020).

### 2.2 Animal husbandry and study design

Using a prospective, randomized intervention design, 64 Sprague–Dawley rats (42 days old; 32 males and 32 females; *n* = 8 per sex per group) were individually housed in Perspex cages (365 × 207 × 140 mm) with metallic mesh lids and wood‐shaving bedding, which was changed twice weekly. The housing environment was maintained under a 12‐h light–dark cycle (07:00–19:00) at 24°C–26°C with adequate ventilation. All rats were fed a commercially sourced rat chow (LabChef, Rodent Breeder, Nutritionhub (Pty) Ltd., Stellenbosch 7602, South Africa) and water ad libitum. They were acclimated for 4 days, during which gelatine containing 0.5% fructose, presented in wide‐mouth glass jars, was introduced into their diet while they adapted to the new cages, holding room environment, and the weighing and feeding regime. Animals were then randomized into four groups: PG (vehicle: gelatine +0.5% fructose), TA (50 mg/kg TA + gelatine +0.5% fructose), SAC (gelatine containing 20% fructose +10% ethanol), and SAC+TA (50 mg/kg TA+SAC gelatine). Fresh preparations were administered daily between 11:00 and 12:00 for 10 weeks.

### 2.3 Preparation of dietary interventions

Dietary interventions were prepared for voluntary oral consumption using gelatine (Sheridan's Gelatine, Libstar, South Africa) as the vehicle. Chemicals used in the preparations included tannic acid (50 mg/kg; CAS 1401‐55‐4; Sigma‐Aldrich, USA), fructose (Nature's Choice, South Africa), and ethanol (99.9%, CAS 64‐17‐5, Batch 37500; ACE Chemicals, South Africa). For Groups 1 (PG) and 2 (TA): 0.5 g fructose was combined with 6 g gelatine in 100 mL warm water. For groups 3 (SAC) and 4 (SAC + TA): 20 g fructose, 6 g gelatine and 10 mL ethanol were mixed in 90 mL water. Tannic acid (0.05 mg/100 mL) was added to the gelatine mixture for groups 2 and 4. Mixtures were stored in sealable wide‐mouthed glass containers and refrigerated at 4°C overnight. Gelatine preparations were administered at a dose of 10 mL/100 g body mass.

### 2.4 Feed intake and body mass

Feed intake was measured weekly by calculating the difference between the amount of feed offered and the feed remaining at the end of each week. Body mass was recorded at baseline, twice weekly throughout the study, and at termination (10 weeks) using a digital balance (Presica 310 M, Precision Instruments, South Africa). Body mass measurements were used to monitor growth and welfare and to adjust the quantity of gelatine and its constituents for each group, ensuring accurate dosing throughout the experiment.

### 2.5 Terminal procedures and assays

#### 2.5.1 Blood collection and analyses

At the end of the 10‐week intervention, rats were fasted overnight. Fasting blood glucose was measured using a glucometer (Bayer Contour Plus, Switzerland) from 0.05 mL of blood obtained via a tail vein prick. Rats were then euthanized by intraperitoneal injection of sodium pentobarbital overdose (200 mg/kg; Elanco Animal Health Pty, South Africa). Following euthanasia, cardiac blood was collected via cardiac puncture using 21G needles into coagulant‐coated tubes and centrifuged at 3000 rpm for 15 min (DLAB DM0412, USA). The resulting serum was aliquoted into microtubes and stored at −80°C for subsequent biochemical and hormone analyses.

#### 2.5.2 Adiposity determination

A midline abdominal incision was performed on each rat to collect visceral adipose tissues, with epididymal fat additionally collected in male rats. Visceral adiposity was assessed by collecting and weighing abdominal fat depots, including mesenteric, perirenal, and retroperitoneal adipose tissues in both male and female rats. These depots were combined to represent total visceral fat for each animal. In male rats, the epididymal fat pads were collected and analyzed separately from the other abdominal fat depots. All fat samples were weighed using an electronic balance, and relative fat mass was calculated as the ratio of visceral or epididymal fat mass to final body weight.

#### 2.5.3 Serum TG, HDL‐C, insulin, and HOMA‐IR

Serum TG and HDL‐C were measured using rat‐specific ELISA kits (TG CAT No: E‐BC‐K261‐M, Detection range: 0.14–10 mmol/L; HDL‐C CAT No: E‐EL‐R0504, Detection range: 3.13–200 ng/mL; Elabscience Biotechnology Co., Ltd, Wuhan, China). TG/HDL‐C ratios were then calculated. Fasting insulin (ELISA kit, CAT No E‐EL‐R3034, Detection range: 6.25–400 pg/mL, Elabscience Biotechnology Co., Ltd, Wuhan, China) and glucose were used to calculate HOMA‐IR (Aref et al., 2013).

$$\text{HOMA}-\mathrm{IR}=\frac{\text{Fasting blood insulin}\;\left(\mathrm{\mu U}/\mathrm{mL}\right)\times \text{Fasting blood glucose}\;\left(\text{mmol}/L\right)}{22.5}$$

#### 2.5.4 Hepatosomatic index and liver storage for assays

Liver lobes were also excised, weighed using a Presica 310M balance, and the hepatosomatic index was subsequently determined using the following formula (Iftikhar et al., 2022).

$$\text{Hepatosomatic index}\;\left(\%\right)=\frac{\text{Absolute liver mass}\;}{\text{Body mass}}\times 100$$

The left lobe was fixed in 4% paraformaldehyde (0.1 M phosphate buffer) at 4°C for histological analysis. The right lobe was preserved in RNAlater™ Stabilization Solution (Invitrogen™/Thermo Fisher Scientific, Cat. No. AM7021, Waltham, MA, USA) at −80°C, and the remaining lobes were snap‐frozen for oxidative stress analyses.

#### 2.5.5 Liver oxidative stress (TBARS) and protein assays

Hepatic lipid peroxidation was assessed by measuring thiobarbituric acid‐reactive substance (TBARS) using a rat‐specific ELISA kit (CAT NO: E‐BC‐K298‐M; Detection range: 2.6–100 μmol/L; Elabscience Biotechnology Co., Ltd, Wuhan, China). Briefly, frozen liver (0.1 g) was thawed, washed in PBS (0.01 M, pH 7.4), sonicated in 1 mL PBS, and centrifuged (10,000 rpm, 5 min, 4°C). Standards and 0.1 mL supernatant were reacted with clarificant and chromogenic agent (3 mL acid + 1 mL TBA), incubated at 95°C–100°C for 60 min, cooled to room temperature, and centrifuged at 1600 rpm for 10 min. Subsequently, 0.25 mL of the supernatant was measured at 532 nm. TBARS concentrations were normalized to total protein, quantified by the Bradford assay (Sigma Aldrich, B6916) using BSA standards (20–1000 μg/mL), with 1:100‐diluted samples, and absorbance read at 595 nm on a microplate reader (Labsystems Multiskan Ascent 354, Thermo Fisher Scientific, Waltham, MA, USA).

#### 2.5.6 Molecular analyses of the liver

Gene expression analysis was performed on RNAlater™‐preserved liver samples (Thermo Fisher Scientific, Waltham, MA, USA). Samples were thawed, lysed in 350 μL lysis buffer supplemented with 3.5 μL β‐mercaptoethanol, and homogenized by sonication. Total RNA was extracted using an RNAspin Mini 96 RNA Isolation kit (Cytiva Life Sciences, Cat. No. 25‐0500‐75, Marlborough, MA, USA). First‐strand cDNA was synthesized using the SuperScript™ IV cDNA Synthesis Kit (Thermo Fisher Scientific, Waltham, MA, USA; Cat. No. 11752‐050) according to the manufacturer's instructions. The mRNA expression of genes related to alcoholic liver disease was quantified by comparative gene expression PCR on a real‐time QuantStudio5 PCR system (Thermo Fisher Scientific, Waltham, MA, USA) using TaqMan probes (ThermoFisher, Carlsbad, USA). Target genes included metabolic markers (*CYP2E1*, *SREBP‐1*) and pro‐inflammatory markers (*NF‐κB1*, *TNF‐α*) and the anti‐inflammatory marker (*IL‐10*). Relative expression levels were calculated using the ΔΔCt method and normalized to β‐actin as the housekeeping gene (Livak & Schmittgen, 2001), as shown in Table 1.

**TABLE 1:** The TaqMan® assay ID of the primers used for comparative gene expression PCR.

Gene (symbol)	Full gene name	Protein product	TaqMan® assay ID
*CYP2E1*	Cytochrome P450 Family 2 Subfamily E Member 1	Cytochrome P450 2E1 enzyme	Rn00580624_m1
*SREBP‐1*	Sterol Regulatory Element‐Binding Protein 1	*SREBP‐1* transcription factor	Rn01495769_m1
*IL‐10*	Interleukin‐10	*IL‐10* cytokine	Rn01483988_g1
*NF‐κB1*	Nuclear Factor kappa‐B subunit 1	*NF‐κB1* p50 protein	Rn01399572_ml
*TNF‐α*	Tumor Necrosis Factor‐alpha	*TNF‐α* cytokine	Rn01525859_g1

#### 2.5.7 Histomorphometry analysis of the liver

Left liver lobes were fixed in 4% paraformaldehyde (0.1 M phosphate buffer, pH 7.4) at 4°C, processed using a tissue processor (Microm STP 120, Thermo Scientific, MA, USA), and embedded in paraffin. Serial sections (5 μm) were cut using a microtome (Leica RM 2125, Wetzlar, Germany) and stained with hematoxylin and eosin (H & E) for morphological evaluation and Masson's Trichrome for collagen detection. Stained slides were scanned using the VS200 digital slide scanner (Evident, USA) and analyzed with QuPath v0.5.1 and ImageJ v1.47v software. Hepatocyte density was determined using 250 × 250 μm counting grids, counting every second grid across 20 grids per section, with QuPath's fast cell count tool (Pohl et al., 2024). Steatosis severity (micro‐ and macrovesicular) was graded from digitized images by blinded observers following the Yip and Burt (2006) classification. Collagen distribution was quantified in ImageJ using color deconvolution (Masson Trichrome vector) and thresholding of color 1 (90–125) to compute the area fraction of collagen relative to a constant total image area (Chen et al., 2017).

### 2.6 Statistical analysis

Data were analyzed using GraphPad Prism version 10 (GraphPad Software, San Diego, USA). Normality was assessed via the Shapiro–Wilk test. For normally distributed data, comparisons across groups were performed using one‐way ANOVA, with results expressed as mean ± SD and Tukey post hoc tests applied where appropriate. Non‐normally distributed data were analyzed using the Kruskal–Wallis test, expressed as median, range or interquartile range (IQR), and Dunn's post hoc test was used for pairwise comparisons. A two‐way ANOVA was performed to evaluate the effects of sex, treatment, and their interaction. Differences were considered statistically significant at *p* < 0.05. Sample numbers vary across analyses because data from animals with PCR results below the assay's detection limit were excluded from comparative gene expression analysis. However, power calculations conducted using OpenEpi (Version 3), based on the mean and standard deviation from a previous study (Zakaria et al., 2025), and assuming a 95% confidence level and 80% power, the resulting sample size was deemed sufficient to achieve adequate power.

## 3 RESULTS

### 3.1 Effect of tannic acid and sweetened alcohol on weekly feed intake and gelatine intake

**FIGURE 1:**
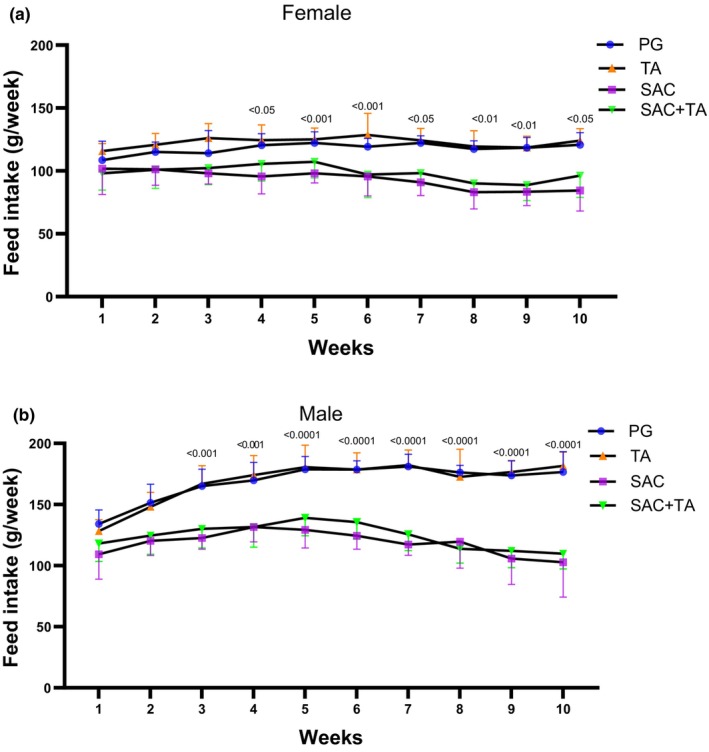
Line graphs illustrating the effect of SAC and TA on the weekly feed intake of (a) the female rats and (b) the male rats. From week 3 to the end of the treatment period, in both sexes, the rats in the SAC and SAC+TA groups had reduced feed intake compared to the PG and TA groups. Data expressed as mean ± Standard deviation, ANOVA, Tukey's post hoc test. ANOVA, analysis of variance; PG, plain gelatine (*n* = 8/sex); TA, tannic acid 50 mg/kg + gelatine (*n* = 8/sex); SAC, 20% fructose + 10% ethanol + gelatine (females *n* = 8, males *n* = 7); SAC+TA, SAC + tannic acid 50 mg/kg (*n* = 8/sex).

Weekly feed intake in female (Figure 1a) and male rats (Figure 1b) was similar across the groups during the first 2 weeks (*p* > 0.05). From week 3 onward, both the SAC and SAC+TA groups consistently exhibited lower feed consumption than the PG and TA groups. In females, feed intake declined by approximately 25% in the SAC group relative to TA at week 3, with significant reductions sustained across weeks 4–10 (SAC/SAC+TA vs. PG/TA, *p* < 0.05). A similar pattern was observed in males beginning at week 3, with lower intake maintained in the SAC and SAC+TA groups throughout the study (*p* < 0.0001). No significant differences were detected between PG and TA, or between SAC and SAC + TA, in either sex (*p* > 0.05).

Absolute and relative weekly gelatine intake (gelatine intake relative to body mass) was similar across groups in both female and male rats throughout the treatment period (*p* > 0.05), as shown in Figure S1.

### 3.2 Effect of tannic acid and sweetened alcohol on body mass

**FIGURE 2:**
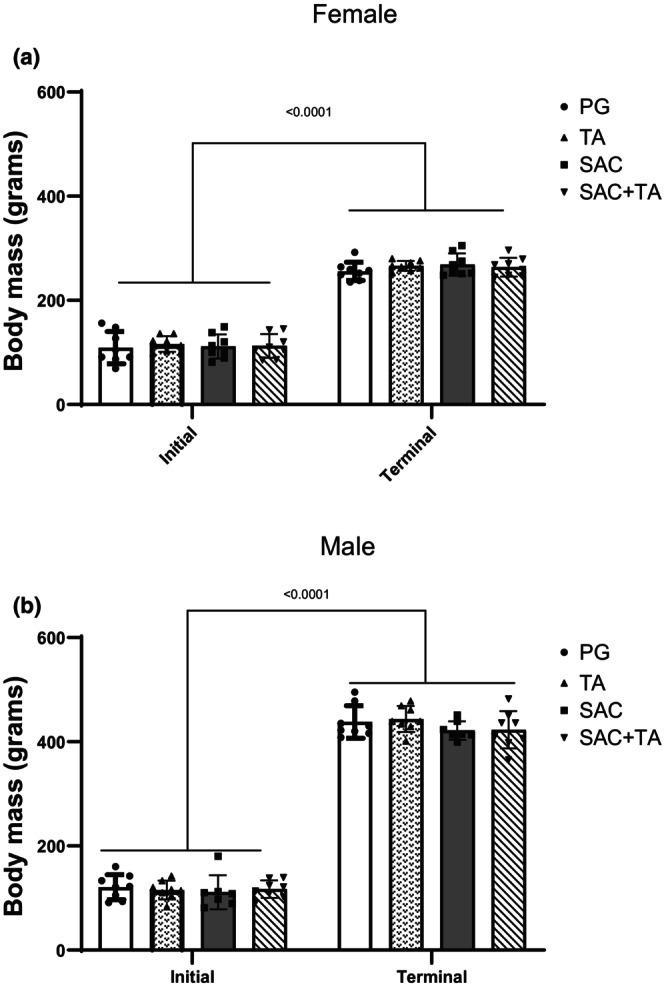
Plots illustrating the effect of SAC and TA on the initial and terminal weights of (a) the female and (b) the male rats. All rats grew significantly across the groups in both sexes. Data expressed as mean ± Standard deviation, ANOVA, Tukey's post hoc test. ANOVA, analysis of variance; PG, plain gelatine (*n* = 8/sex); TA, tannic acid 50 mg/kg + gelatine (*n* = 8/sex); SAC, 20% fructose + 10% ethanol + gelatine (females *n* = 8, males *n* = 7); SAC+TA, SAC + tannic acid 50 mg/kg (*n* = 8/sex).

Initial body mass was similar across groups in both sexes (*p* > 0.05). All groups showed significant body mass gain from baseline to the end of the study (*p* < 0.0001). However, no differences in body mass were observed between treatment groups at either time point (initial and terminal) (*p* > 0.05). At termination, females weighed significantly less than males across all treatments (sex effect: *p* < 0.001; treatment effect: *p* = 0.5219; interaction: *p* = 0.2515) (Figure 2).

### 3.3 Effect of tannic acid and sweetened alcohol on fat accumulation

In female rats, both absolute and relative visceral fat differed significantly across treatment groups (*p* = 0.0374 and *p* = 0.0468, respectively); however, Dunn's post hoc tests revealed no significant pairwise differences. In the male rats, there was no significant difference in the absolute and relative visceral fat (absolute: *p* = 0.8636; relative: *p* = 0.7278) and epididymal fat (absolute: *p* = 0.4524; relative: *p* = 0.9874) across the groups. When comparing sexes, females exhibited higher relative visceral fat than males in the TA (*p* = 0.0082), SAC (*p* = 0.0302), and SAC+TA (*p* = 0.0060) groups, but not in the PG (*p* = 0.585). Overall, both sex and treatment effects were significant (*p* < 0.0001 and *p* = 0.0399, respectively), with no significant interaction effect (*p* = 0.4537) (Table 2).

**TABLE 2:** A summary of the mean and median values of the absolute and relative visceral fat and epididymal fat for the female and the male rats across the different experimental groups.

	PG		TA		SAC		SAC + TA		*p*
	Mean ± SD	Median	Mean ± SD	Median	Mean ± SD	Median	Mean ± SD	Median	
Female (F)									
Visceral fat (g)	6.73 ± 0.83	6.63	8.79 ± 1.40	8.84	8.85 ± 3.05	7.59	9.64 ± 3.07	9.76	0.0374*
Visceral fat (% BW)	2.63 ± 0.30	2.58	3.31 ± 0.55^*#*^	3.24	3.25 ± 0.84^*$*^	2.97	3.64 ± 1.11^*€*^	3.76	0.0468*
Male (M)									
Visceral fat (g)	8.90 ± 1.78	8.79	9.28 ± 2.68	8.90	9.10 ± 2.13	8.90	10.09 ± 3.09	10.21	0.8636
Visceral fat (% BW)	2.03 ± 0.38	1.96	2.76 ± 0.51^*#*^	1.92	2.15 ± 0.43^*$*^	2.17	2.38 ± 0.68^*€*^	2.37	0.7278
Epididymal fat (g)	2.77 ± 0.32	3.90	2.67 ± 0.58	3.50	2.56 ± 0.56	3.30	2.59 ± 0.39	3.25	0.4524
Epididymal fat (% BW)	0.63 ± 0.09	0.64	0.60 ± 0.11	0.58	0.60 ± 0.11	0.57	0.61 ± 0.09	0.60	0.9874

*Note*: For each parameter, *indicates a significant difference across the treatment groups at *p* < 0.05, Kruskal–Wallis test, Dunn's test, and ANOVA test for Epidydimal fat (%BW). When the females were compared to the males in the same group, the same superscript symbols (#, $, and €) indicate a significant difference at *p* < 0.05 using a Two‐way Analysis of variance followed by Tukey's post hoc test.

Abbreviations: ANOVA, analysis of variance; PG, plain gelatine (F = 8, M = 8); SAC, 20% fructose, 10% alcohol and gelatine (F = 8, M = 7); SAC+TA, 20% fructose, 10% alcohol, Tannic acid (TA‐50mg/kg) and gelatine (F = 8, M = 8); SD, standard deviation; TA, tannic acid (TA‐50mg/kg) and plain gelatine (F = 8, M = 8).

### 3.4 Effect of tannic acid and sweetened alcohol on fasting blood glucose, insulin, and HOMA‐IR

Fasting blood glucose (FBG) levels were similar across the groups in both females (*p* = 0.0519) and males (*p* = 0.0816), except for higher FBG in females than males within the TA group (*p* = 0.0388). Overall, sex and treatment effects on FBG were significant (sex *p* = 0.0007; treatment *p* = 0.0834), with no significant interaction effect (*p* = 0.0652). Serum insulin concentrations did not differ across treatment groups in either females (*p* = 0.6530) or males (*p* = 0.8517), and no significant main or interaction effects were observed (sex: *p* = 0.3349; treatment: *p* = 0.7627; interaction: *p* = 0.6691). Similarly, HOMA‐IR values were similar across the treatment groups in both sexes (female: *p* = 0.3005; male: *p* = 0.3021) with no significant effects of sex (*p* = 0.0720), treatment (*p* = 0.9411), or sex vs. treatment interaction (*p* = 0.0736) (Table 3).

**TABLE 3:** A summary of the mean values of the fasting blood glucose, the insulin, and the HOMA‐IR for the female and male rats across the different experimental groups.

	PG		TA		SAC		SAC+TA		*p*
	Mean ± SD	Median	Mean ± SD	Median	Mean ± SD	Median	Mean ± SD	Median	
Female (F)									
Fasting blood Glucose (mmol/L)	3.73 ± 0.35	3.70	4.14 ± 0.35^*#*^	4.10	3.80 ± 0.50	3.60	3.61 ± 0.24	3.60	0.0519
Insulin (pg/mL)	401.60 ± 93.97	399.10	385.80 ± 92.44	380.90	422.50 ± 79.72	388.40	448.10 ± 97.81	484.30	0.6530
HOMA‐IR	1.67 ± 0.27	1.69	2.00 ± 0.52	1.97	2.07 ± 0.59	1.88	2.07 ± 0.48	2.22	0.3005
Male (M)									
Fasting blood Glucose (mmol/L)	3.78 ± 0.55	3.90	3.43 ± 0.53^*#*^	3.50	3.16 ± 0.58	3.30	3.33 ± 0.31	3.25	0.4424
Insulin (pg/mL)	406.60 ± 110.60	366.70	393.80 ± 80.52	363.80	367.00 ± 32.55	374.10	397.80 ± 80.21	403.10	0.8517
HOMA‐IR	1.97 ± 0.65	1.73	1.76 ± 0.55	1.65	1.48 ± 0.19	1.51	1.67 ± 0.27	1.69	0.3021

*Note*: For each parameter, no significant differences were observed across treatment groups in either sex (*p* > 0.05), based on the One‐Way Analysis of variance, except for fasting blood glucose (female), which was analyzed using Kruskal–Wallis test. When the females were compared to the males in the same group, the same superscript symbols (#) indicate a significant difference at *p* < 0.05 using a Two‐way Analysis of variance followed by Tukey's post hoc test.

Abbreviations: SD, standard deviation; PG, plain gelatine (F = 8, M = 8); TA, TA‐50 mg/kg and plain gelatine (F = 8, M = 8); SAC, 20% fructose, 10% alcohol and gelatine (F = 8, M = 7); SAC+TA, 20% fructose, 10% alcohol, TA‐50 mg/kg and gelatine (F = 8, M = 8).

### 3.5 Effect of tannic acid and sweetened alcohol on serum TG, HDL‐C, and TG/HDL‐C ratio

Serum TG levels did not differ across treatment groups in either females (*p* = 0.3332) or males (*p* = 0.1990). A significant sex effect was observed (*p* = 0.0006) with no significant treatment (*p* = 0.1757) or sex and treatment interaction effects (*p* = 0.3318). HDL‐C levels were unchanged across treatment groups in females (*p* = 0.9326). In males, HDL‐C levels were significantly higher in the SAC (*p* = 0.0405) and SAC+TA (*p* = 0.0312) groups compared with the PG group, indicating that TA did not modify the SAC‐induced increase in HDL‐C. No significant sex, treatment, or interaction effects were detected for HDL‐C (sex: *p* = 0.6872; treatment: *p* = 0.1251; interaction: *p* = 0.2241). TG/HDL‐C ratios were similar across groups in both females (*p* = 0.3931) and males (*p* = 0.8517), although a significant sex effect was present (*p* = 0.0035), with no treatment (*p* = 0.8396) or interaction (*p* = 0.1132) effects (Table 4).

**TABLE 4:** A summary of the mean values of the serum TG, HDL‐C, and TG‐HDL‐C ratio for the female and the male rats across the different experimental groups.

	PG	TA	SAC	SAC + TA	*p*
	Mean ± SD	Mean ± SD	Mean ± SD	Mean ± SD	
Female (F)					
TG (mmol//L)	0.49 ± 0.22	0.74 ± 0.37	0.75 ± 0.39	0.72 ± 0.39	0.3332
HDL‐C (mmol//L)	0.34 ± 0.06	0.36 ± 0.10	0.35 ± 0.10	0.33 ± 0.08	0.1441
TG/HDL‐C	1.50 ± 0.71	2.06 ± 0.83	2.30 ± 1.42	2.21 ± 0.88	0.3921
Male (M)					
TG (mmol//L)	0.93 ± 0.25	0.82 ± 0.22	1.02 ± 0.42	1.20 ± 0.46	0.1994
HDL‐C (mmol//L)	0.29 ± 0.08^ab^	0.33 ± 0.08	0.47 ± 0.20^a^	0.39 ± 0.08^b^	0.0175*
TG/HDL‐C	3.45 ± 1.36	2.62 ± 1.05	2.34 ± 1.56	3.07 ± 1.23	0.3108

*Note*: Row means with the same superscript letters (a, b) indicate a significant difference at *p* < 0.05, ANOVA, Tukey's post hoc test.

Abbreviations: SD, standard deviation; PG, plain gelatine (F = 8, M = 8); TA, TA‐50 mg/kg and plain gelatine (F = 8, M = 8); SAC, 20% fructose, 10% alcohol and gelatine (F = 8, M = 7); SAC+TA, 20% fructose, 10% alcohol, TA‐50 mg/kg and gelatine (F = 8, M = 8).

### 3.6 Effect of tannic acid and sweetened alcohol on liver mass and hepatosomatic index

Absolute liver mass did not differ across treatment groups in either females (*p* = 0.3615) or males (*p* = 0.7204). Similarly, the hepatosomatic index remained unchanged in females (*p* = 0.4739) and males (*p* = 0.1026). A significant sex effect was observed for both liver mass and hepatosomatic index (*p* < 0.0001), while no significant treatment (liver mass: *p* = 0.5177; hepatosomatic index: *p* = 0.1189) or sex versus treatment interaction effects were detected (liver mass: *p* = 0.5874; hepatosomatic index: *p* = 0.7803) (Table 5).

**TABLE 5:** A summary of the mean values of the liver mass and hepatosomatic index for the female and the male rats across the different experimental groups.

	PG	TA	SAC	SAC+TA	*p*
	Mean ± SD	Mean ± SD	Mean ± SD	Mean ± SD	
Female (F)					
Liver mass (g)	7.63 ± 0.57^*#*^	8.00 ± 0.62^$^	8.33 ± 1.06^*€*^	8.24 ± 0.95^*Δ*^	0.3615
Body mass (g)	255.80 ± 17.73	266.30 ± 9.27	268.60 ± 21.57	263.90 ± 18.01	0.4823
Hepatosomatic index (%)	2.99 ± 0.19	3.01 ± 0.19	3.09 ± 0.19	3.13 ± 0.24^#^	0.4739
Male (M)					
Liver mass (g)	12.00 ± 1.07^*#*^	12.00 ± 0.76^$^	12.16 ± 0.84^*€*^	11.97 ± 1.24^*Δ*^	0.7204
Body mass (g)	438.00 ± 31.36	443.80 ± 24.77	421.70 ± 17.73	422.90 ± 35.85	0.3500
Hepatosomatic index (%)	2.74 ± 0.11	2.82 ± 0.12	2.88 ± 0.09	2.83 ± 0.11^#^	0.1026

*Note*: For each parameter, there was no difference across the treatment groups in both sexes of rats, *p* > 0.05 using One‐Way Analysis of variance. When the females were compared to the males in the same group, the same superscript symbols (#, $, €, and Δ) indicate a significant difference at *p* < 0.05 using a Two‐way Analysis of variance followed by Tukey's post hoc test.

Abbreviations: SD, standard deviation; PG, plain gelatine (F = 8, M = 8); TA, TA‐50 mg/kg and plain gelatine (F = 8, M = 8); SAC, 20% fructose, 10% alcohol and gelatine (F = 8, M = 7); SAC+TA, 20% fructose, 10% alcohol, TA‐50 mg/kg and gelatine (F = 8, M = 8).

### 3.7 Effect of tannic acid and sweetened alcohol on liver TBARS

**FIGURE 3:**
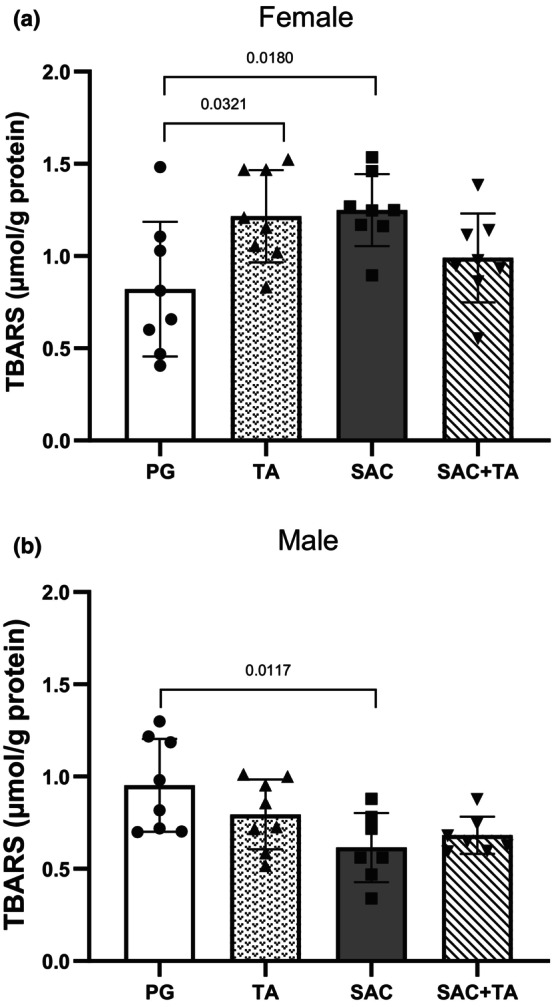
Plots showing the effect of SAC and TA on TBARS concentration in the liver for (a) the female and (b) the male rats. The female rats in the TA and SAC groups had higher TBARS concentrations than the PG group, while the SAC group had lower TBARS concentrations compared to the PG group in the male rats. Data expressed as mean ± standard deviation, ANOVA, Tukey's post hoc test. ANOVA, analysis of variance; PG, plain gelatine (*n* = 8/sex); TA, tannic acid 50 mg/kg + gelatine (*n* = 8/sex); SAC, 20% fructose + 10% ethanol + gelatine (females *n* = 8, males *n* = 7); SAC+TA, SAC + tannic acid 50 mg/kg (*n* = 8/sex).

In females (Figure 3a), TBARS concentrations differed significantly across treatment groups (*p* = 0.0111). Compared with the PG group, TBARS levels were elevated in the TA group (*p* = 0.0321) and the SAC group (*p* = 0.0180). In contrast, the SAC+TA group in female rats showed TBARS concentrations comparable to the PG group (*p* = 0.6972). In males (Figure 3b), TBARS also differed across groups (*p* = 0.0122), with SAC showing lower TBARS levels than the PG (*p* = 0.0117), while TA (*p* = 0.3786) and SAC + TA (*p* = 0.0535) did not differ from PG. A significant sex effect (*p* < 0.0001) and sex vs. treatment interaction (*p* = 0.0003) were observed, although the treatment effect was not significant (*p* = 0.2403).

### 3.8 Effect of TA and SAC on hepatic mRNA expression of metabolic and inflammatory markers

As shown in Table 6, neither TA nor SAC significantly altered the hepatic mRNA expression of metabolic (*CYP2E1* and *SREBP‐1*) or inflammatory (*IL‐10*, *NF‐κB1*, and *TNF‐α*) markers in either sex (*p* > 0.05, ANOVA). There were also no significant sex‐, treatment‐, or interaction‐related effects for *CYP2E1* (Sex: *p* = 0.1890; Treatment: *p* = 0.2360; Interaction: *p* = 0.2390), *SREBP‐1* (Sex: *p* = 0.3182; Treatment: *p* = 0.1812; Interaction: *p* = 0.9298), *IL‐10* (Sex: p = 0.1890; Treatment: *p* = 0.2361; Interaction: p = 0.2390), *NF‐κB1* (Sex: *p* = 0.4768; Treatment: *p* = 0.0983; Interaction: *p* = 0.9425), or *TNF‐α* (Sex: *p* = 0.5917; Treatment: *p* = 0.5520; Interaction: *p* = 0.6159).

**TABLE 6:** A summary of the mean and median values of the mRNA expression levels of some genes in the liver of the female and the male rats across the different experimental groups.

mRNA expression	PG		TA		SAC		SAC + TA		*p*
	Mean ± SD	Median	Mean ± SD	Median	Mean ± SD	Median	Mean ± SD	Median	
Female (F)									
*CYP2E1*	0.93 ± 0.16	0.95	1.02 ± 0.39	1.06	1.20 ± 0.86	0.81	0.92 ± 0.55	0.68	0.1224
*IL‐10*	1.20 ± 0.83	0.99	1.21 ± 0.67	1.13	0.77 ± 0.28	0.88	0.73 ± 0.54	0.62	0.5590
*NF‐κB1*	1.03 ± 0.27	0.99	1.02 ± 0.22	1.08	1.01 ± 0.33	1.07	0.75 ± 0.11	0.75	0.3535
*SREBP‐1*	1.17 ± 0.70	1.20	1.26 ± 0.59	1.16	1.09 ± 0.49	1.12	0.63 ± 0.34	0.50	0.4030
*TNF‐α*	1.03 ± 0.28	1.05	1.04 ± 0.23	1.08	1.30 ± 0.73	1.20	1.48 ± 0.80	1.47	0.4191
Male (M)									
*CYP2E1*	1.57 ± 0.41	1.37	1.88 ± 0.69	2.12	1.08 ± 0.48	1.05	0.72 ± 0.86	0.53	0.7113
*IL‐10*	1.24 ± 0.81	1.25	0.59 ± 0.30	0.56	0.51 ± 0.04	0.53	0.43 ± 0.19	0.43	0.5175
*NFκB1*	1.02 ± 0.23	0.94	1.13 ± 0.36	1.12	1.14 ± 0.26	1.18	0.79 ± 0.14	0.79	0.2571
*SREBP‐1*	1.13 ± 0.63	1.02	0.89 ± 0.41	0.74	0.92 ± 0.19	0.99	0.46 ± 0.11	0.46	0.3966
*TNF‐α*	1.04 ± 0.36	1.07	1.03 ± 0.52	1.17	1.47 ± 0.45	1.03	0.87 ± 0.49	1.02	0.4848

*Note*: For each parameter, no significant differences were observed across treatment groups in either sex (*p* > 0.05), based on the One‐Way Analysis of variance, except for CYP2E1 female, IL‐10 male, and TNF‐α female, which were analyzed using the Kruskal–Wallis test.

Abbreviations: CYP2E1, cytochrome P450 2E1; IL‐10, interleukin‐10; NF‐κB1, nuclear factor kappa B1; PG, plain gelatine (F = 4, M = 4); SAC, 20% fructose, 10% alcohol and gelatine (F = 4, M = 3); SAC+TA, 20% fructose, 10% alcohol, TA‐50mg/kg and gelatine (F = 4, M = 2); SD, standard deviation; SREBP‐1, sterol regulatory element‐binding protein‐1; TA, TA‐50mg/kg and plain gelatine (F = 4, M = 4); TNF‐α, tumour necrosis factor‐α.

### 3.9 Effect of TA and SAC on liver cell density

**FIGURE 4:**
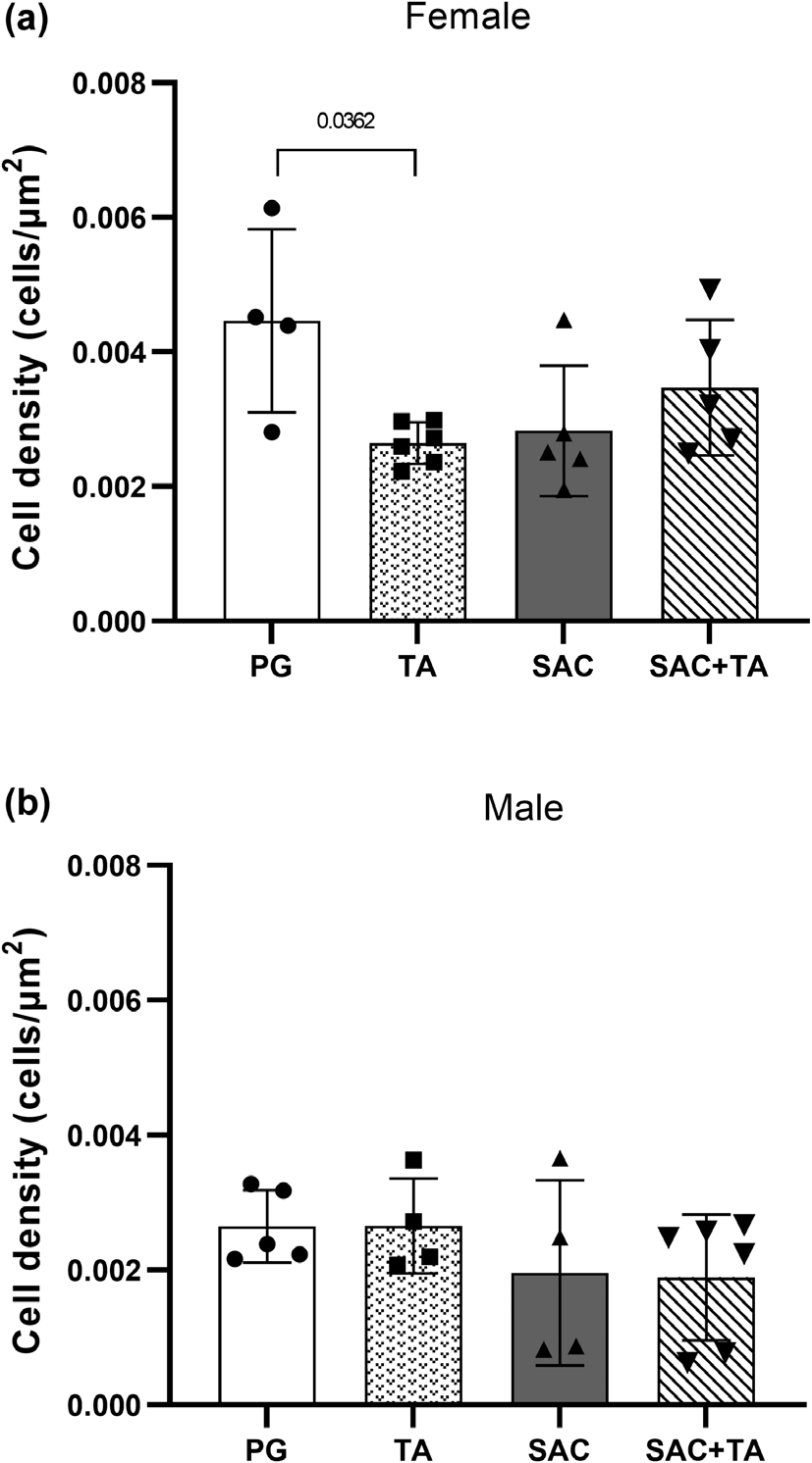
Bar graphs showing the effect of SAC and TA on the liver cell density of (a) the female and (b) the male rats. The female rats in the TA group had a lower cell density than the PG group, whereas there was no significant difference in cell density across treatment groups in male rats. Data are presented as mean and standard deviation, and Tukey's post hoc test. PG, plain gelatine (females *n* = 4, males *n* = 5); TA, TA‐50 mg/kg and plain gelatine (females *n* = 6, males *n* = 4); SAC, 20% fructose, 10% alcohol and gelatine (females *n* = 5, males *n* = 4); SAC+TA, 20% fructose, 10% alcohol, TA‐50 mg/kg and gelatine (females *n* = 5, males *n* = 7), Average number of grids assessed per rat ≈34, Average Grid Area = 62,518.81μm^2^.

As shown in Figure 4, liver cell density differed significantly among the female groups overall (*p* = 0.0385). Post hoc analysis showed that TA reduced liver cell density compared with the PG group (*p* = 0.0362). In contrast, SAC and SAC+TA groups did not differ significantly from PG (*p* = 0.0788 and *p* = 0.4106, respectively). In males, liver cell density did not differ significantly among the groups (*p* = 0.4168). Across groups, females had lower liver cell density than males, with a significant sex effect (*p* = 0.0013); treatment and interaction effects were not significant (*p* = 0.0632 and *p* = 0.1533, respectively).

### 3.10 Effect of TA and SAC on hepatic steatosis scores

**FIGURE 5:**
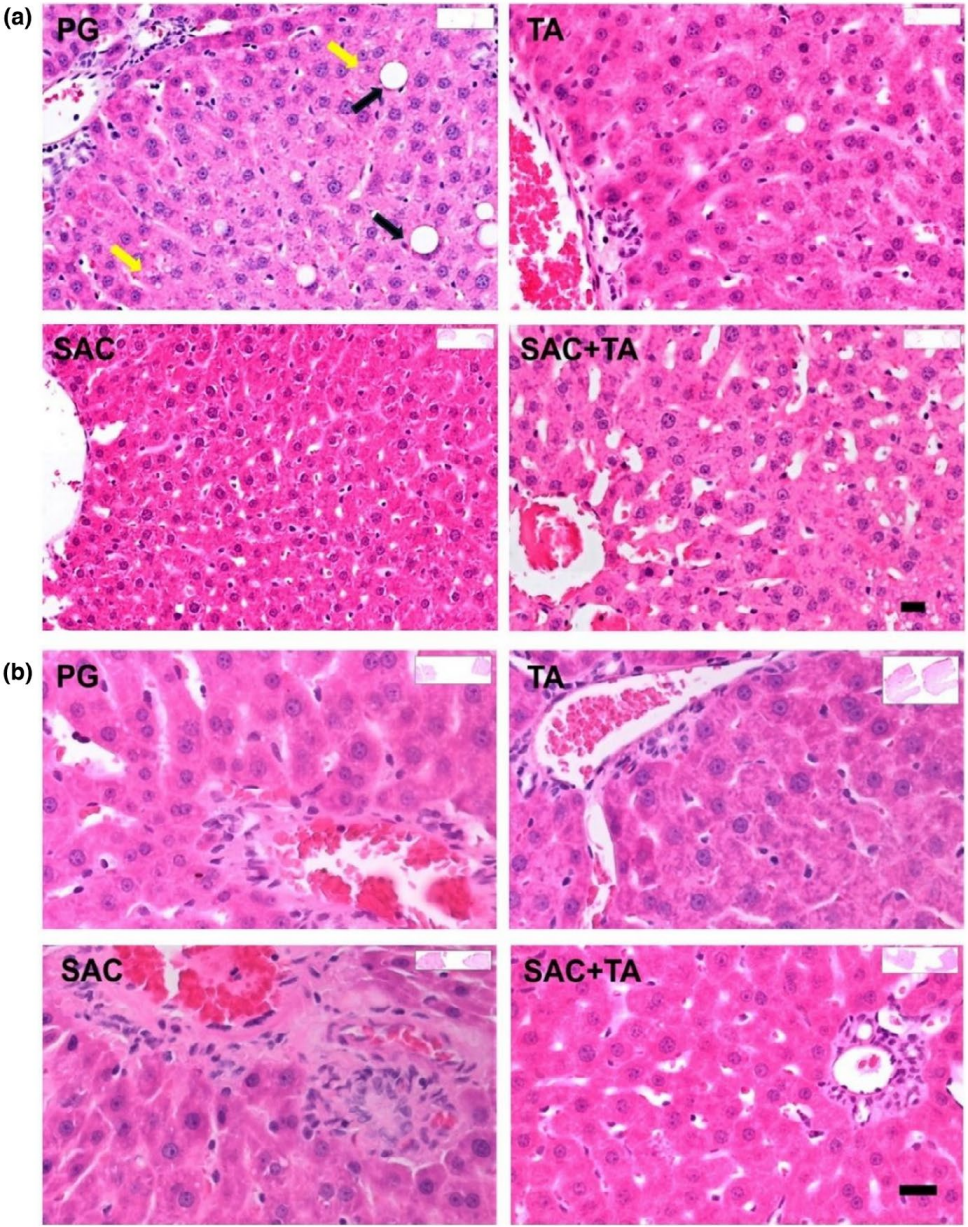
Representative photomicrographs of the liver of (a) the female and (b) the male rats. The sections were stained with Hematoxylin and Eosin. The yellow arrows indicate microsteatosis, while the black arrows indicate macrosteatosis in the liver. Scale bar = 20 μm. Image at the top right corner = section map, PG, plain gelatine; TA, TA‐50 mg/kg and plain gelatine; SAC, 20% fructose, 10% alcohol and gelatine; SAC+TA, 20% fructose, 10% alcohol, TA‐50 mg/kg and gelatine.

The effects of TA and SAC on steatosis scores are summarized in Table 7, with representative H&E‐stained liver sections shown in Figure 5. In females, neither microvesicular (*p* = 0.4022) nor macrovesicular (*p* = 0.3985) steatosis differed across treatment groups. In males, microvesicular steatosis was significantly elevated in the SAC group compared with both PG (*p* = 0.0088) and SAC+TA (*p* = 0.0043), whereas macrovesicular steatosis remained unchanged (*p* = 0.6057). Significant sex effects were observed for both microvesicular (*p* < 0.0001) and macrovesicular steatosis (*p* = 0.0024), with females generally exhibiting higher scores than males, except in the SAC group, where males showed elevated microvesicular steatosis.

**TABLE 7:** A summary of the median and interquartile range values of the steatosis scores for the females and the males across the different experimental groups.

	PG	TA	SAC	SAC+TA	*p*
	Median (Q1–Q3)	Median (Q1–Q3)	Median (Q1–Q3)	Median (Q1–Q3)	
Female (F)					
Micro vesicular steatosis	2 (1–3)^#^	1 (0–2)^*€*^	2 (0–2)	2 (0.25–3)^*Δ*^	0.4022
Macro vesicular steatosis	1 (0–1)^$^	0 (0–0.75)	0 (0–0)	0 (0–1)^#^	0.3985
Male (M)					
Micro vesicular steatosis	0 (0–1)^ab#^	0 (0–1)^*€*^	1 (0.25–1.75)^a^	1 (0–2)^b *Δ*^	0.0013
Macro vesicular steatosis	0 (0–0)^$^	0 (0–0)	0 (0–0)	0 (0–0)^#^	0.6057

*Note*: For each parameter, the same superscript letters (a, b, c, and d) indicate a significant difference between any two groups at *p* < 0.05 using Kruskal–Wallis followed by Dunn's post hoc test. The same superscript symbols (#, $, €, and Δ) indicate a significant difference at p < 0.05 when the females were compared to the males in the same group using a Two‐way Analysis of variance followed by Tukey's post hoc test.

Abbreviations: PG, plain gelatine (no of images assessed, *F* = 15, M = 18); Q1–Q3, interquartile range; SAC, 20% fructose, 10% alcohol and gelatine (no of images assessed, F = 18, M = 12); SAC+TA, 20% fructose, 10% alcohol, TA‐50mg/kg and gelatine (no of images assessed, F = 12, M = 19); TA, TA‐50mg/kg and plain gelatine (no of images assessed, F = 12, M = 14).

### 3.11 Effect of TA and SAC on collagen distribution

**FIGURE 6:**
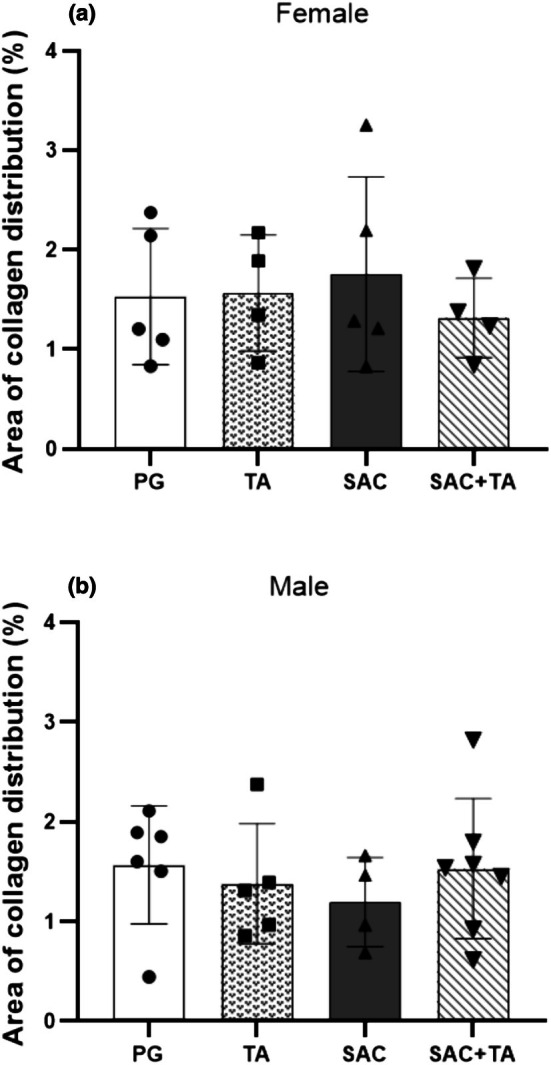
Bar graphs showing the effect of SAC and TA on the percentage area of liver collagen distribution for (a) the female and (b) the male rats. In both sexes of rats, there was no difference in the percentage area of collagen distribution. Data are presented as mean and standard deviation, ANOVA, and Tukey's post hoc test. PG, plain gelatine (females *n* = 5, males *n* = 5); TA, TA‐50 mg/kg and plain gelatine (females *n* = 4, males); SAC, 20% fructose, 10% alcohol and =20% fructose (females *n* = 5, males *n* = 4); SAC+TA, 20% fructose, 10% alcohol, TA‐50 mg/kg and gelatine (females *n* = 4, males *n* = 7), Average number of images assessed per rat ≈13.

**FIGURE 7:**
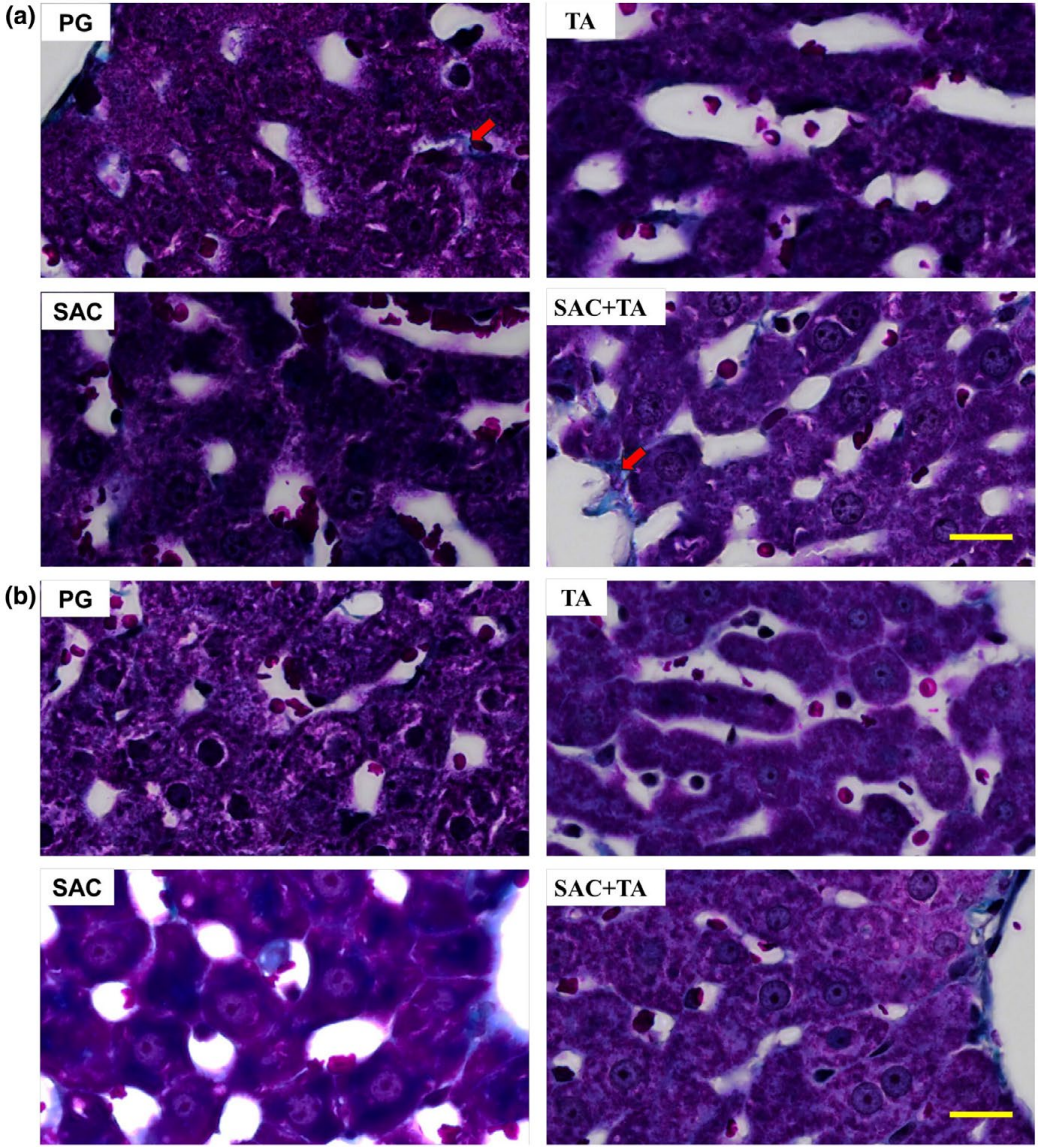
Representative photomicrographs of the liver of (a) the female and (b) the male rats. The sections were stained with Masson trichrome. The orange arrows indicate some areas of collagen in the liver. Scale bar = 20 μm. PG, plain gelatine; TA, TA‐50 mg/kg and plain gelatine; SAC, 20% fructose, 10% alcohol and gelatine; SAC+TA, 20% fructose, 10% alcohol, TA‐50 mg/kg and gelatine.

The effects of TA and SAC on collagen distribution are shown in Figure 6, with representative Masson trichrome‐stained sections in Figure 7. In both females and males, the collagen‐positive area did not differ across groups (Females: *p* = 0.8395; males: *p* = 0.7778). Across sexes, there were no significant sex differences (*p* = 0.5619), treatment effect (*p* = 0.9781), and interaction effect (*p* = 0.6175).

## 4 DISCUSSION

This study examined the effects of voluntary consumption of tannic acid (TA) and sweetened alcohol (SAC) on metabolic and hepatic health in adolescent male and female Sprague–Dawley rats.

### 4.1 Feed intake and body mass

SAC was associated with reduced feed intake, which may reflect the appetite‐suppressive effects of alcohol and fructose reported in previous studies. Alcohol enhances leptin and cholecystokinin signaling, reducing hunger (Bach et al., 2021; Richardson et al., 1990), while fructose increases leptin secretion and dampens hypothalamic feeding drive (de Oliveira et al., 2020). Although alcohol is energy‐dense (7 kcal/g) and nutrient‐poor (Brenes et al., 2021), and fructose provides 3.6 kcal/g (Mattar et al., 2022), comparable to rat chow, the reduced intake may reflect compensatory adjustments in energy intake (Kwon et al., 2024). Despite lower feed intake, SAC did not affect body mass, aligning with reports that moderate alcohol consumption does not consistently impact growth in rodents (Rasineni et al., 2021). While high‐fructose diets can increase adiposity at higher doses or longer durations (Kumar et al., 2021), the moderate fructose level and reduced feed intake associated with alcohol exposure may have contributed to the absence of detectable weight gain. TA alone did not alter feed intake, contrasting with studies showing appetite stimulation at higher doses (Chung et al., 2019). The low TA concentration (0.005%) used here may explain this discrepancy. Additionally, fructose may have diminished the perceived bitterness of tannic acid (Osakabe et al., 2024), which could potentially have reduced taste aversion and facilitated its consumption. However, because feed intake during the acclimatization period was not recorded (as the rats were provided the same type of feed and quantities), these intake patterns must be interpreted with caution, as potential baseline differences between groups were not assessed. Overall, moderate SAC was associated with reduced feed intake without detectable changes in growth or adiposity under the present experimental conditions, while low‐dose TA showed minimal observable effects on feeding behavior.

### 4.2 Visceral adiposity

Visceral and epididymal fat mass were unaffected by TA or SAC, aligning with reports that moderate alcohol intake does not consistently promote visceral fat and may even reduce fat accumulation (Rasineni et al., 2021; Zhang et al., 2015). In contrast, high‐fructose diets are known to increase abdominal obesity (Ramos et al., 2017). The absence of detectable changes in visceral adiposity may suggest that metabolic alterations can occur before overt fat accumulation, as reported in previous fructose‐feeding studies (Kovačević et al., 2021), and may also reflect age‐related metabolic demands during adolescence (Ghasemi et al., 2021). Notably, females exhibited higher relative visceral fat across most groups, these findings may be consistent with known hormonal influences on fat distribution (Zong et al., 2020).

### 4.3 Glucose and lipid metabolism

Neither SAC nor TA affected fasting glucose, insulin, or HOMA‐IR, suggesting no detectable impairment in insulin sensitivity under the present experimental conditions. This aligns with findings that moderate alcohol (Rasineni et al., 2016) and sugar intake (de Oliveira et al., 2020) do not significantly disrupt glycaemic control, though prolonged high‐sugar diets remain diabetogenic (Veit et al., 2022). Triglycerides and TG/HDL‐C ratios were unchanged, suggesting no early signs of dyslipidaemia. Notably, HDL‐C was elevated in SAC‐fed males, consistent with the known HDL‐raising effects of moderate alcohol (Kwon et al., 2024) and fructose (Benado et al., 2004). This effect was absent in females, highlighting sex‐specific lipid responses. The increase in HDL‐C in males may partly explain the observed reduction in hepatic TBARS, given HDL's antioxidant and anti‐inflammatory roles (Denimal, 2024).

### 4.4 Oxidative stress and hepatic function

A key finding was the sex‐specific response to oxidative stress. Female rats showed elevated hepatic TBARS following SAC and TA. The increase observed following SAC is consistent with reports describing greater female susceptibility to alcohol‐induced lipid peroxidation (Lonappan et al., 2024). The elevation observed in the TA‐only group may reflect the context‐dependent pro‐oxidant activity that has been reported for certain polyphenols, including tannic acid, under specific experimental conditions (Mhlanga et al., 2019). In contrast, SAC reduced TBARS in males, potentially linked to an increase in HDL‐C. Notably, TA co‐administration normalized TBARS in both sexes, consistent with a potential antioxidant effect of TA, possibly through mechanisms previously reported in the literature, including modulation of ROS‐generating enzymes such as glutathione S‐transferase and NQO1 (Gülçin et al., 2010; Karakurt & Adalı, 2011). These findings suggest that the oxidative effects associated with TA may vary according to the underlying metabolic environment and sex.

Despite differences in oxidative stress, hepatic *CYP2E1* and *SREBP‐1* expression remained unchanged. *CYP2E1* is typically upregulated by chronic high‐dose alcohol exposure (Sandoval et al., 2022), while *SREBP‐1* responds to high fructose or alcohol intake (Ferré et al., 2021; Yu et al., 2022). Their unchanged expression indicates that *CYP2E1* and *SREBP‐1* mRNA levels were not altered under the experimental conditions, suggesting oxidative stress in the absence of detectable changes in *CYP2E1* and *SREBP‐1* mRNA expression, possibly mediated by alternative regulators such as Nrf2 or PPARα (Q. Li et al., 2023). However, this does not exclude potential involvement of these pathways at the protein or functional level, which were not assessed in this study.

Inflammatory gene expression (*IL‐10*, *NF‐κB1*, and *TNF‐α*) was also unaffected, consistent with studies using low‐to‐moderate alcohol or fructose exposures (Kołota et al., 2020; Melo et al., 2021). Higher doses are known to activate *NF‐κB* and increase pro‐inflammatory cytokines (Iskender et al., 2022; Li et al., 2018). The absence of inflammatory activation aligns with preserved liver mass and hepatosomatic index.

### 4.5 Hepatic histological outcomes

Histological analysis revealed sex‐specific vulnerability to hepatic injury, with female rats exhibiting lower liver cell density than males across groups. In females, TA treatment reduced liver cell density compared with the PG group, whereas SAC and SAC+TA treatments did not differ significantly from controls. In males, liver cell density was not significantly altered by treatment. Histological examination also revealed more pronounced micro‐ and macrovesicular steatosis in females, suggesting greater susceptibility to hepatic lipid accumulation compared with males, consistent with reports describing enhanced female vulnerability to alcohol‐ and fructose‐associated liver injury (Hyer et al., 2019; Kono et al., 2000; Li et al., 2019). Potential mechanisms may involve estrogen‐mediated Kupffer cell activation, increased caspase activity, and sex differences in alcohol metabolism (Li et al., 2019; Sayaf et al., 2022).

In males, SAC‐induced microsteatosis without progression to macrosteatosis may reflect early‐stage fatty liver changes. Elevated HDL‐C may be associated with reduced lipid accumulation (Hamaguchi et al., 2012). Collagen distribution did not differ significantly among treatment groups in either sex. These findings suggest that although histological evidence of steatosis was present, SAC and TA exposure did not substantially alter hepatic collagen deposition within the experimental period. Nevertheless, previous studies have reported antifibrotic effects of TA through suppression of stellate cell activation and collagen crosslinking (Baldwin & Booth, 2022; Chu et al., 2016), as well as context‐dependent effects of moderate alcohol exposure on fibrosis progression (Sun et al., 2018).

This study acknowledges several important limitations. First, the absence of fluid‐intake measurements restricted our ability to assess hydration status and its potential influence on metabolic outcomes, thereby limiting the interpretation of fluid balance–related effects. Second, feed intake was recorded only during the experimental period and not during the pre‐treatment acclimatization phase. As a result, feed‐intake values could not be normalized to baseline, and therefore, the comparative interpretation of energy intake across groups should be approached with caution. Baseline consumption patterns may influence subsequent metabolic responses, and the inability to account for this variation represents a meaningful constraint. Similarly, the inability to determine the total energy density of the formulation due to the unavailability of calorific values for the gelatine vehicle and the sweetened alcohol component is acknowledged as a limitation of the study. Consequently, the reported analysis does not account for the full energy contribution of these constituents, which may affect the precision of the overall energy estimation.

Additionally, the lack of hepatic lipid profiling and leptin quantification reduced our capacity to fully characterize lipid metabolism, adiposity‐related signaling, and appetite‐regulatory mechanisms. Another limitation of this study is that interpretations regarding the involvement of *CYP2E1* and *SREBP‐1* are based on gene expression data alone. As mRNA levels do not necessarily reflect protein abundance or functional activity, the absence of transcriptional changes does not exclude their potential involvement in oxidative stress at the protein or post‐translational level. Future studies would benefit from incorporating these biochemical assessments, as well as exploring additional molecular pathways, including pro‐apoptotic regulators and other endocrine mediators, to more comprehensively elucidate the mechanisms underlying the sex‐specific responses observed in this investigation.

## 5 CONCLUSION

Collectively, these findings suggest sex‐dependent responses to SAC under the present experimental conditions. Female rats exhibited higher TBARS levels, reduced hepatocyte density, and more pronounced steatosis under the present experimental conditions, consistent with clinical evidence of greater female susceptibility to alcohol‐related liver injury (Eagon & Mandell, 2010). In contrast, males exhibited patterns associated with elevated HDL‐C and reduced hepatic TBARS levels. TA supplementation attenuated oxidative stress in both sexes, consistent with potential antioxidant and antifibrotic effects, though it did not fully mitigate female vulnerability.

These findings have important translational implications for adolescent health. With the increasing popularity of sweetened alcoholic beverages, often marketed as “Jello shots”, adolescents are exposed to combined alcohol and sugar intake during a critical developmental window. Our findings indicate that such exposures may be associated with early hepatic changes, even in the absence of overt weight gain or dyslipidaemia, with females showing greater susceptibility to oxidative and steatotic changes in this model. While TA may warrant further investigation as a dietary polyphenol with potential protective effects, its efficacy may be limited by sex‐specific vulnerabilities. Therefore, public health strategies aimed at reducing adolescent alcohol and sugar consumption remain essential, and nutraceuticals like TA warrant further exploration as supplementary interventions.

## AUTHOR CONTRIBUTIONS

**Toluwase E. Olanipekun:** Data curation; formal analysis; funding acquisition; investigation; methodology; visualization. **Oladiran I. Olateju:** Conceptualization; data curation; formal analysis; funding acquisition; investigation; methodology; project administration; resources; software; supervision; validation; visualization. **Monica Gomes:** Data curation; formal analysis; funding acquisition; investigation; methodology; validation; visualization. **Ashmeetha Manilall:** Data curation; formal analysis; funding acquisition; investigation; methodology; validation; visualization. **Kennedy H. Erlwanger:** Conceptualization; data curation; formal analysis; funding acquisition; investigation; methodology; project administration; resources; supervision; validation; visualization.

## FUNDING INFORMATION

This work was supported by the Department of Physiology Research Incentive Funds, University of the Witwatersrand (001.169.8521101.5121.105.000000.0000000000.4463), the NRF Thuthuka (TTK210301588226) and CSUR (CSUR240320210064) grants, and the Faculty of Health Sciences Research Committee Individual Research Grant, University of the Witwatersrand.

## CONFLICT OF INTEREST STATEMENT

The authors declare that they have no conflict of interest.

## ETHICS STATEMENT

The study was granted ethical clearance (AREC/2022/11/06C) from the Animal Research Ethics Committee at the University of the Witwatersrand, Johannesburg. All experiments were carried out at the Wits Research Animal Facility (WRAF) at the University of the Witwatersrand, in accordance with the South African National Standard for animal care and use for scientific purposes (SANS 10386, 2021).

## Supporting information


Figure S1.

## Data Availability

This study is based on TEO's PhD thesis, which was submitted to the University of the Witwatersrand in 2025 (Olanipekun, 2025). The thesis is currently not available online but can be made available upon request. The dataset supporting this manuscript is available in the Wits University Open Data Vault with the following DOI: https://doi.org/10.71796/wits‐figshare.32625705. The dataset contains all measured data presented in this manuscript for each animal, identified by a unique animal ID. The data include weekly feed intake, weekly gelatine intake, body mass, visceral fat mass, fasting glucose, insulin, HOMA‐IR, lipid profiles, and hepatic outcomes. Hepatic outcomes include liver mass, TBARS, gene expression data, and histological features.
