# Supplementary material for: Sex‐specific hepatic effects of sweetened alcohol consumption and tannic acid intervention in adolescent rats
Source: Physiol Rep. 2026 Jun 30;14(13):e70992. doi: 10.14814/phy2.70992 (PMC13315809; doi:10.14814/phy2.70992)
Supplement: Supplementary file 1 — Figure S1. [file PHY2-14-e70992-s001.docx]

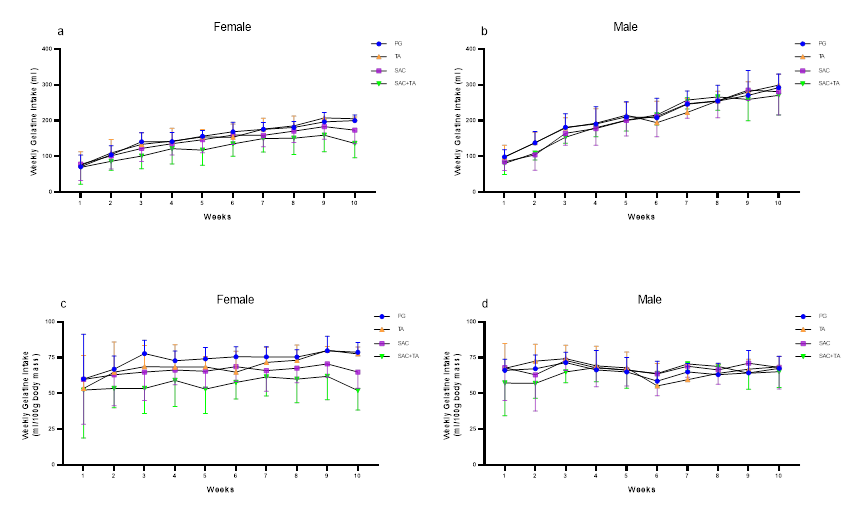


Figure S1: Line graphs illustrating the effects of SAC and TA on weekly gelatine intake. Graphs (a) and (b) show the absolute gelatine intake in female and male rats, respectively, while graphs (c) and (d) show the gelatine intake relative to body mass in female and male rats, respectively. When compared across groups at each week, gelatine intake remained comparable throughout the study period in both sexes of rats. *Data expressed as mean ± Standard deviation, p>0.05, ANOVA, Tukey’s post hoc test. ANOVA = Analysis of variance, PG = plain gelatine (n = 8 females, 8 males), TA = Tannic acid-50mg/kg and plain gelatine (n = 8 females, 8 males), SAC = 20% fructose, 10% alcohol and gelatine (n =8 females, 7 males), SAC+TA = 20% fructose, 10% alcohol, TA-50mg/kg and gelatine (n =8 females, 8 males).*
